# Poisoning of workers working in small lead-based units

**DOI:** 10.4103/0019-5278.44697

**Published:** 2008-12

**Authors:** Harsiddha G. Sadhu, B. K. Amin, D. J. Parikh, N. G. Sathawara, Umesh Mishra, B. K. Virani, B. C. Lakkad, V. K. Shivgotra, Shruti Patel

**Affiliations:** National Institute of Occupational Health, Meghani Nagar, Ahmedabad - 380 016, India; 1 Department of Medicine, B J Medical College, Civil Hospital, Ahmedabad - 380 016, India

**Keywords:** Lead poisoning, small-scale industry, treatment, workers

## Abstract

**Background:**

No data are available with the labor departments among the workers of small-scale lead-based units with regard to lead poisoning. One hundred and ninety-five workers were investigated for lead exposure and three were found exceeding the limit of 80 mg/dL, which required a treatment for lead poisoning.

**Aim:**

To assess the exposure and health risk in workers working in small lead-based units.

**Setting and Design:**

Random sampling is selected from the cross-sectional medical study.

**Methods and Materials:**

Medical examination cum biochemical/hematological investigations along with blood lead estimation were carried out in these workers.

**Statistical Analysis:**

Epi-Info and SPSS 16.0 were used for statistical analysis.

**Results and Conclusion:**

Workers' blood lead levels were brought down from 114.4, 110.0 and 120.6 mg/dL with treatment of D-penicillamine to 40 mg/dL. It may be concluded that lead poisoning is a preventable public health problem that particularly affects the industrial workers in small lead-based units.

## INTRODUCTION

Lead poisoning is one of the compensable diseases in India since 1924.[1] However, reliable statistics and scientific data regarding occurrence of occupational lead poisoning are not available with the respective departments.

One hundred and ninety-five workers working in small-scale lead battery manufacturing, lead stearate, lead oxide and battery recycling plants in Ahmedabad and nearby areas were included. Workers are exposed to lead particulates and fumes during the process through inhalation and ingestion of dust due to poor housekeeping and unhygienic conditions. Six workers had blood lead levels > 80μg/dL. As per the DG FASLI,[2] blood lead levels > 80 μg/dL needs treatment; hence, attempts were made for treatment.

## MATERIALS AND METHODS

Six workers from the 195 studied were found to have blood lead levels exceeding 80 *μ*g/dL, of which three were from battery recycling units, two from a lead oxide plant and one from a lead stearate plant. The workers from the battery recycling units did not cooperate for treatment, whereas the remaining three workers were given treatment.

The study encompasses medical cum biochemical/hematological investigations along with lead estimation in blood samples of these three subjects. The physician of the Civil Hospital, Ahmedabad, carried out the medical examination. Biochemical tests like blood sugar, serum glutamic pyruvate transaminase serum glutamic oxaloacetate transaminase, serum alkaline phosphates, cholesterol, creatinine, urea, bilirubin, serum protein, potassium, calcium, etc., using an auto analyzer with standard biochemical methods, the erythrocyte sedimentation rate and basophilic stippling using standard methods and the chest X-ray were carried out in the Civil Hospital. Hematological parameters such as hemoglobin, total count and differential count using a cell counter (Sysmax-KH21) and lead levels in the blood analyzed using an atomic absorption spectrophotometer (Perkin Elmer, Model 3100) were carried out at the NIOH along with the quality control samples to assure the validity of the results.

## RESULTS

The first, second and third workers aged 41, 22 and 44 years, respectively, had more than 8, 4 and 6 years past experience in the lead manufacturing (oxide and sterate) units, respectively.

All the three workers were exposed to lead dust and fume during their work and were not using any personal protective equipment (mask, gloves and boots). In addition, the first two workers were also likely to ingest lead dust during and off the shift as they were staying in the plant premises.

Nothing significant was elucidated in the past, family or personal history of all the cases. The first worker had a habit of smoking, drinking alcohol and tobacco chewing, the second had a habit of drinking alcohol and tobacco chewing while the third had a habit of tobacco chewing only.

**Figure 1:**
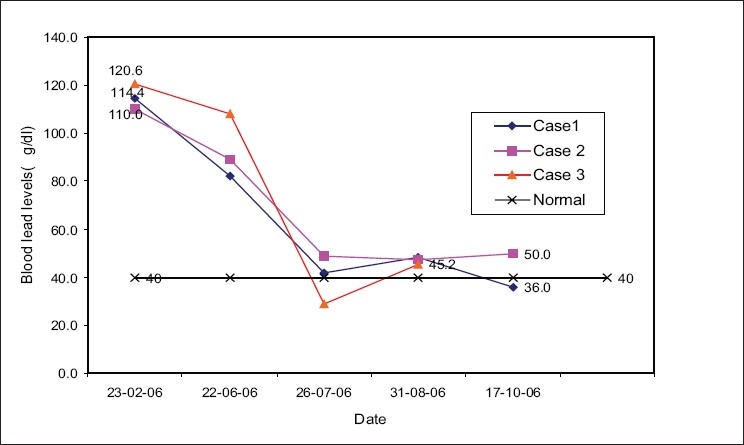
Blood Lead Levels in Cases

In all the three workers, the vitals were normal, with pulse rates of 80/min and a blood pressure of 120/80, 128/88 and 110/80 mm Hg, respectively. The first worker had pain in the right knee and was unable to move while working. The second had abdominal pain in both the flanks whereas the third worker had anorexia, pain and burning in both feet at the time of examination. The results of hematology, electro cardiogram, chest X-ray, routine urine examination and biochemical investigations for these cases were within the normal range, indicating normal liver, kidney and heart functions. Their blood lead levels were markedly elevated [Figure 1]. They were given oral D-penicillamine (250 mg) four times a day for 15 days and advised not to work in the plant during this time. The medical examination, investigations and blood lead levels were carried out periodically in all the cases. Figure 1 shows that the lead levels were reduced remarkably with treatment over the study period. In the first case, the last lead level was 36.0 μg/dL. In the other two, the lead levels were slightly higher than 40 μg/dL. It is observed in the third case that the lead level was reduced to 29 μg/dL at the time of the third estimation but then increased again, and the last lead level reported in this case was 50 μg/dL. This could possibly be due to the release of lead from the bones and/or resuming the work, although rest in the hospital was strongly recommended to all these workers.

All the three workers did not cooperate for further follow-up for the treatment. Generally, it is difficult to get the cooperation in small-scale units due to various reasons such as lack of education and awareness and loss of wages during the participation. In this case, with great effort, a reasonably good cooperation was obtained.

## DISCUSSION

It is observed that the engineering controls are installed in battery oxide and lead sterate plants but these controls are not effective and are not maintained regularly. Because of a lack of proper awareness/education related to lead poisoning and not using the effective personal protective devices, these workers are exposed to lead dust and fumes and do not wear proper and clean clothing during the work.

Out of the three workers studied, two stayed in the factory premises where lead oxide was being manufactured. Thus, the workers remain prone to a heath risk for all the 24 h. After the treatment with D-penicillamine, the lead levels are decreased; however, they did not come down to normal levels (< 40 μg/dL) in two workers [Figure 1]. These workers were also recommended to remain away from the work place but due to their economic constrain, lack of employment opportunities and social conditions, and the desire to work with the same job only, these workers did not follow the advice of temporarily stopping the work in the lead factory. According to Staudinger and Roth,[3] blood lead levels less than 40 mg/dL are acceptable for chronic exposure to lead, but workers should be tested every 6 months. The South African Lead Regulation[4] stipulates that blood lead levels should not exceed 60 mg/dL and should always remain less than 40 mg/dL.

It may be concluded that lead poisoning is an important and preventable health problem that particularly affects the industrial workers working in small units and the children of low socioeconomic status. It is therefore essential to workout a national policy related to this issue taking into account the cost benefit analysis.[6]
